# Regulation of dopamine transporter activity by carboxypeptidase E

**DOI:** 10.1186/1756-6606-2-10

**Published:** 2009-05-06

**Authors:** Heping Zhang, Shupeng Li, Min Wang, Brian Vukusic, Zdenek B Pristupa, Fang Liu

**Affiliations:** 1 Department of Neuroscience, Centre for Addiction and Mental Health, Toronto, Ontario, Canada; 2 Departments of Psychiatry, University of Toronto, Toronto, Ontario M5T 1R8, Canada

## Abstract

**Background:**

The dopamine transporter (DAT) plays a critical role in terminating the action of dopamine by rapid reuptake into the presynaptic neuron. Previous studies have revealed that the DAT carboxyl terminus (DAT-CT) can directly interact with other cellular proteins and regulate DAT function and trafficking.

**Results:**

Here, we have identified that carboxypeptidase E (CPE), a prohormone processing exopeptidase and sorting receptor for the regulated secretory pathway, interacts with the DAT-CT and affects DAT function. Mammalian cell lines coexpressing CPE and DAT exhibited increased DAT-mediated dopamine uptake activity compared to cells expressing DAT alone. Moreover, coexpression of an interfering DAT-CT minigene inhibited the effects of CPE on DAT. Functional changes caused by CPE could be attributed to enhanced DAT expression and subsequent increase in DAT cell surface localization, due to decreased DAT degradation. In addition, CPE association could reduce the phosphorylation state of DAT on serine residues, potentially leading to reduced internalization, thus stabilizing plasmalemmal DAT localization.

**Conclusion:**

Taken together, our results reveal a novel role for CPE in the regulation of DAT trafficking and DAT-mediated DA uptake, which may provide a novel target in the treatment of dopamine-governed diseases such as drug addiction and obesity.

## Background

The dopamine transporter (DAT) is a presynaptic plasma protein found on dopaminergic nerve terminals that terminates dopamine signaling by rapidly sequestering dopamine released into the synaptic cleft [1,2]. DAT belongs to a family of Na+/Cl- dependent transporters with several other neurotransmitter transporters, including norepinephrine, serotonin, GABA, and glycine transporters [2-4]. Dopamine (DA), an essential neurotransmitter for normal CNS functions including motor activity, cognition and reward [5,6], which is involved in numerous neurological disorders including Parkinson's disease, drug addiction and schizophrenia, is primarily cleared from the synapse [7]. Moreover, the DAT has garnered attention because it is the principle target of widely abused psychostimulants, such as cocaine and amphetamine [2-4].

Morphological studies reveal that functional DATs are localized on the plasma membrane of axons, pre-synaptic axon terminals, and dendrites of dopaminergic neurons, with the highest expression in the axonal processes in striatum and dendrites in ventral tegmental area [8-10]. Specifically, immunoelectron microscopy studies find that DAT is located peri-synaptically, indicating that transmitter released at the synapse diffuses out of the cleft to be transported into the terminal [9,11]. Thus, specific targeting and presentation of DAT in neurons require efficient recycling of transporters after internalization or efficient export of newly synthesized transporters from the endoplasmic reticulum (ER), sorting to transport carriers in the Golgi complex and delivery/retention of transporters at the presynaptic functional sites. The expression, trafficking and function of DAT are regulated by several factors including DAT substrates, inhibitors, and a variety of signaling cascades including protein kinases and phosphatases [12-14]. Moreover, structure-function analysis reveals several important amino acid residues or structural motifs critical for DAT function. The carboxyl terminus (CT) of human DAT has been demonstrated to be important for cell surface expression and proper function of DAT [7,15,16]. Recent studies using yeast two-hybrid approaches have successfully identified several proteins that interact with the DAT carboxyl terminus and influence DAT trafficking [16-18]. The PDZ domain-containing protein PICK1, which binds to a type II PDZ binding site localized at the DAT distal carboxyl termini, increases the efficiency of ER-to-plasma membrane trafficking of the transporter [7,16]. Conversely, Hic-5, a LIM domain containing protein, has also been shown to bind to the DAT-CT and inhibit DAT localization to the plasma membrane [17]. In addition, our lab has shown that the DAT CT can also bind to α-synuclein, which stabilizes DAT on the surface and could accelerate DA-induced oxidative stress and cell death [18].

The increasing reports of proteins that interact with DAT [7,19-26] and their roles in the modulation of physiological and pathological DAT functions stress the importance to understand the molecular mechanisms of how these interaction proteins affect DAT metabolic cycle and trafficking. Here, in an effort to search for candidate proteins capable of interacting with DAT and affecting DAT trafficking, we identified the protein carboxypeptidase E (CPE). Biochemical, immunological, and functional approaches were employed to examine assembly and trafficking properties of DAT with CPE.

## Methods

### Yeast two-hybrid screening and β-galactosidase assays

PCR was used to generate CPE-activating domain (AD) fusion constructs in either pACT2 or pGADT7 and binding domain(BD) fusion constructs with the DAT intracellular amino-terminal (NT: Met1-Lys66), carboxyl-terminal (CT1: Leu583-Val620), and carboxyl-terminal fragments (CT2: Glu598-Val620; CT3: Leu583-Pro597) in pAS2 or pGBKT7. Appropriate pairs of AD and BD constructs were cotransformed into the yeast strain Y187 grown at 30°C for 3 days on Leu-/Trp- medium lacking leucine and tryptophan, followed by assay of lacZ reporter activation, present downstream of a GAL4 binding sequence in Y187, as described by the manufacturer (Clontech). All control transformations were negative for β-galactosidase activity except for BD/p53 with AD/T antigen, used as a positive control. All constructs were sequenced to determine appropriate splice fusion and absence of spurious PCR-generated mutations.

### Coimmunoprecipitation, affinity purification (pull-down), and Western blotting

Coimmunoprecipitation, affinity pull-down, and Western blot analyses were performed as previously described [27-29]. Rat brain striatum (100 mg) or transfected COS-7 cells (~2 × 107) were homogenized in buffer containing (in mM) 50 Tris-Cl, pH 7.6, 150 NaCl, 2 EDTA, 1 PMSF plus 1% Igepal CA-630, 0.5 to ~1% sodium deoxycholate, 1% Triton X-100, and protease inhibitor mixture (Sigma); after being centrifuged at 10,000 × g at 4°C for 20 min, the supernatant was extracted and protein concentrations were measured (Pierce, Rockford, IL). For coimmunoprecipitation experiments, solubilized striatum/cell extracts (500 to 700 μg of protein) were incubated in the presence of primary antibodies anti-CPE (Chemicon, B.D Transduction Laboratories™), anti-DAT (Santa Cruz Biotechnology, Santa Cruz, CA), or rabbit IgG (1 to 2 μg) for 4 hr at 4°C, followed by the addition of 20 μl of protein A/G agarose (Santa Cruz Biotechnology, Santa Cruz, CA) for 12 hr at 4°C. Pellets were washed four times in the buffer described above, boiled for 5 min in SDS sample buffer, and subjected to SDS-PAGE. In each experiment 20 to 50 μg of tissue-extracted protein was used as control. For affinity purification experiments the solubilized striatum extracts (50–100 μg of protein) were incubated with glutathione-Sepharose beads (Pharmacia, Dorval, Québec, Canada) bound to the indicated GST-fusion proteins (50 to 100 μg) at 4°C for 12 hr. Beads were washed three times with 600 μl of PBS containing 0.1–0.5% Triton X-100 before the bound proteins were eluted with glutathione elution buffer. Elutes were incubated in sample buffer and subjected to SDS-PAGE for Western blot analysis. Blots were blocked with 5% nonfat dried milk dissolved in TBST buffer (10 mM Tris, 150 mM NaCl, and 0.1% Tween-20) for 1 hr at room temperature, washed three times with TBST buffer, and then incubated with the appropriate primary antibody (anti-DAT or anti-CPE, diluted in 1% milk in TBST) overnight at 4°C and washed again with TBST buffer three times; the membrane was incubated with horseradish peroxidase-conjugated secondary antibody (diluted in 1% milk in TBST; Sigma) for 1.5 hr at room temperature. The proteins were visualized with enhanced chemiluminescence reagents as described (Amersham Biosciences).

### In vitro binding assay

A probe was made with CPE subcloned into mammalian expression vector pCDNA3 using TNT^® ^T7 Quick Coupled Transcription/Translation System (Promega, Madison, WI) and [^35^S] methionine (PerkinElmer, Waltham, MA). Glutathione beads carrying 20 μg GST-DAT-NT, GST-DAT-CT or GST alone were incubated at room temperature for 1 hour with [^35^S] methionine-labeled CPE probe, respectively. The beads were then washed six times with PBS containing 0.1–0.5% (V/V) Triton X-100 and eluted with 10 mM glutathione elution buffer. Eluates were separated by SDS-PAGE and visualized by autoradiography using BioMax (Kodak) film.

### [^3^H] Dopamine uptake analysis and [^3^H] WIN binding

We measured dopamine uptake on intact cells as described previously [18]. Two to 4 days after transfection in 24-well plates (~2 × 10^5 ^cells seeded per well), medium was removed and wells were rinsed with 0.5 ml of uptake buffer (5 mM Tris, 7.5 mM HEPES, 120 mM NaCl, 5.4 mM KCl, 1.2 mM CaCl2, 1.2 mM MgSO4, 1 mM ascorbic acid, 5 mM glucose, pH7.1). Cells were then preincubated in duplicate with the indicated concentrations of dopaminergic agents (10^-13 ^to 10^-4^M) 5 min before the addition of 0.25 ml of 20 nM [^3^H]dopamine (final concentration) and incubated for 10 min at room temperature in a total volume of 0.5 ml. Nonspecific [^3^H] dopamine (37–53 Ci/mmol) uptake was defined in the presence of 10 μM GBR-12909. Wells were rinsed twice with 0.5 ml of uptake buffer; cells were solubilized in 0.5 ml of 1%SDS and collected to measure incorporated radioactivity using a Beckman liquid scintillation counter (LS 6000SC). For [^3^H] WIN binding, the differences from uptake are using [^3^H] WIN (4 nM final concentration) and incubating for 2 to 3 h at 4°C before adding 1% SDS.

### Cell-ELISA assays

Cell-ELISA assays (colorimetric assays) were done essentially as previously described [22,29]. HEK-293 cells were transiently transfected with the indicated cDNA constructs by the Lipofectamine method (6–10 μg of each indicated cDNA per 7.5 × 10^6 ^cells), distributed equally to two six-well plates (35 mm/well), and grown for 2–4 d. The cotransfected cells were fixed in 2% paraformaldehyde for 10 min in the absence (nonpermeabilized conditions) or the presence (permeabilized conditions) of 1% TritonX-100. Cells were incubated with anti-DAT (Chemicon) for the purpose of labeling the receptors on the cell surface under nonpermeabilized conditions or the entire receptor pool under permeabilized conditions. After incubation with the corresponding horseradish peroxidase (HRP)-conjugated secondary antibodies (Sigma), the HRP substrate o-phenylenediamine (OPD; Sigma) was added to produce a color reaction that was stopped with 3 N HCl. The cell surface and total expression of DAT was presented as the colorimetric readings under nonpermeabilized and permeabilized conditions and then normalized to the control groups (transfected with DAT alone) respectively.

### Pulse-Chase analysis

HEK 293 T cells were transiently transfected with DAT or together with CPE, grown to near-confluency over 12 hr, pre-incubated in extracellular solution (7 mM HEPES, 121 mM NaCL, 3 mM NaHCO3, 1 mM Na-pyruvate, 1.8 mM CaCL2, 20 mM glucose, 0.01 mM glycine, 5 mM KCL), for 1 h at 37°C, and labeled in fresh medium containing 0.2 mCi/ml promix [35S] methionine/cysteine (Amersham Biosciences). After incubation for a further 4 h at 37°C, the pulse was terminated by washing cells twice and incubating with MEM (supplemented with 10% FBS) for the indicated times. Immunoprecipitation of DAT was carried out.

### Cell culture and confocal imaging

After tranfection (as above) for 2–4 d, HEK-293 cells were used for double-label immunoconfocal microscopy. CPE mouse Abs (Pharmacia) and DAT Rabbit Abs (Santa Cruz) were used as primary antibodies in overnight incubations at 4°C and detected using secondary antibodies (Sigma) conjugated to FITC or Cy3 fluorophores. Control cells in which no primary Ab was included did not reveal any immunoreactive staining.

### GST fusion proteins and mini-gene

DAT CT cDNA-encoding fragment was amplified by PCR from full-length cDNA clones [18]. DAT CT and DAT NT GST-fusion proteins were prepared from bacterial lysates as described by the manufacturer (Amersham Biosciences). To confirm appropriate splice fusion and the absence of spurious PCR-generated nucleotide errors, we resequenced all constructs.

### Acute Rat Striatum slices

Acute hippocampal slices (350 μm) were prepared from Sprague-Dawley rats using a McIlwain tissue chopper (Mickle Laboratory Engineering, Gomshall, UK). Under deep anesthesia, brains were rapidly removed and striatum was dissected out and leave for 5 mins in ice-cold artificial cerebrospinal fluid (ACSF) containing (mM) NaCl, 126; KCl, 2.5; MgCl2, 1; CaCl2, 1; KH2PO4, 1.25; NaHCO3, 26; and glucose, 20 that was bubbled continuously with carbogen (95%O2/5%CO2) to adjust the pH to 7.4. Freshly cut slices were placed in an incubating chamber with carbogenated ACSF and recovered from stress at 37°C for 1 hr. Slices were then treated with/without PMA for 30 mins and harvested for Co-immunoprecipitation and Western blotting analysis.

## Results

### DAT CT directly interacts with CPE

**Figure 1:**
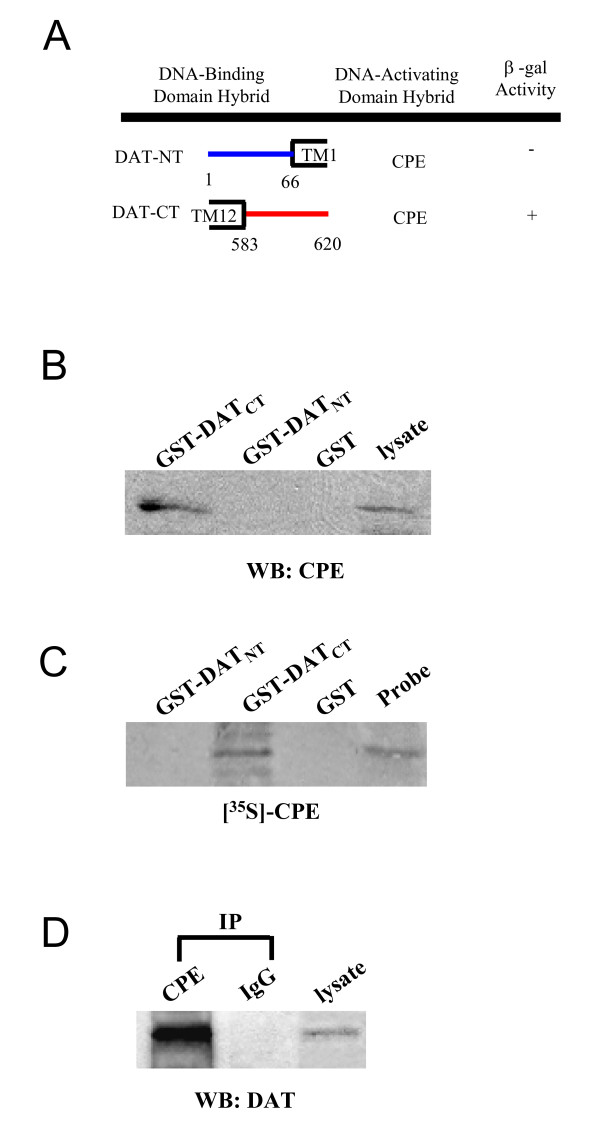
**Association of DAT with CPE**. **A**, Using the Yeast 2-Hybrid System 3 (Invitrogen), 1.7 × 106 independent clones of a human substantia nigra library (in pGAD10) were screened using DAT-NT, containing the amino terminus up to the first transmembrane domain (TM1) and DAT-CT, containing the entire carboxyl terminus after the 12th transmembrane domain (TM12) as baits. Interacting clones were selected on media lacking Leu, Trp, and His, in the presence of 2.5 mM 3-AT. Of 158 colonies growing under this selection, 35 were positive for lacZ expression. Sequence analysis of these clones showed that two of them represented fragments of carboxypeptidase E (CPE). One clone showing very strong lacZ expression encoded a portion of CPE beginning at amino acid residue 123 of the mature protein. No interaction was observed between CPE construct and Gal4 BD fused to murine p53 or human lamina C or between any of the DAT-BD fusions and Gal4 AD fused to SV40 large T antigen. **B**, GST fusion protein precipitation assay using the complete C and N terminus of DAT fused to GST. Aliquots containing GST fusion fragments were incubated with rat striatal extracts and analyzed by Western blot using a polyclonal anti-CPE antibody. **C**, Using an *in vitro *binding assay, [^35^S]-CPE probe bound with GST-DAT-CT, but not with GST-DAT-NT or GST alone. **D**, DAT coimmunoprecipitated with CPE antibodies from rat striatal extracts.

To search for DAT-interacting proteins, we employed the entire DAT-CT as bait to screen a human substantia nigra library using the yeast two-hybrid system (Fig 1A). Interacting clones were selected on Leu-/Trp- media. Of the 158 colonies growing under this selection, 35 were positive for lacZ expression. Sequence analysis revealed that 2 of the 3 clones were fragments of CPE, which was previously described as a sorting receptor to target prohormones to the secretory granules of the regulated secretory pathway in neuroendocrine cells [30].

To further confirm the interaction between DAT and CPE, we performed GST affinity pull down assay using purified GST fusion peptides containing sequences from DAT-CT (GST-DAT-CT). The results shown in Fig. 1B demonstrated that GST-DAT-CT could selectively precipitate CPE from solubilized rat striatal tissue whereas GST alone was unable to purify CPE. Moreover, to exclude the possibility of CPE non-specifically binding to GST fusion peptides, intracellular N-terminal domain of DAT was also employed as a control. As shown in Fig 1B, GST-DAT-NT was unable to precipitate CPE, strongly suggesting that the interaction between DAT and CPE is mediated specifically by the DAT-CT. While these results demonstrate the presence of the DAT: CPE protein complex in rat striatal tissue, it does not clarify whether the DAT: CPE protein complex is formed through a direct interaction or is mediated indirectly by an accessory binding protein. *In vitro *binding assays were performed to provide evidence that DAT and CPE can directly interact with each other. As shown in Figure 1C, *in vitro *translated [^35^S]-CPE probe hybridized with GST-DAT-CT but not GST-DAT-NT or GST alone, indicating the specificity of the direct protein-protein interaction between CPE and DAT-CT. Taken together, these data suggest that CPE is involved in a direct protein-protein interaction with DAT-CT.

Next, we examined in solubilized rat striatum, whether DAT could coimmunopreicipiate with CPE thus providing additional evidence of a physical association between DAT and CPE. As illustrated in Fig. 1D, CPE antibodies co-precipitated a protein with a relative molecular mass of 83 kDa that immunoreacted with DAT antibodies. Taken together, these results showed that DAT could associate with CPE via the DAT-CT.

### Coexpression CPE and DAT increased the Vmax of DAT without effects on its affinity

**Figure 2:**
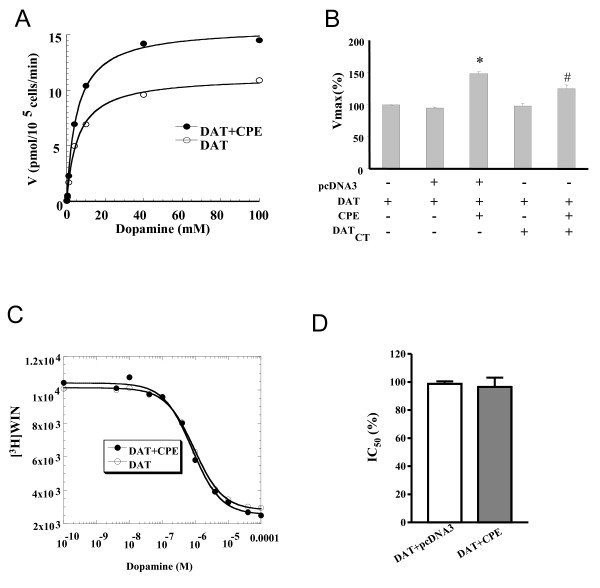
**CPE-mediated enhancement of [^3^H] DA uptake**. **A**, Representative saturation plots of [^3^H]DA uptake in HEK-293 cells expressing the DAT alone or coexpressing-CPE revealed an increase in the *Vmax *for [^3^H] DA uptake accumulation (n = 9, P < 0.01) with no significant alteration in estimated *Km *values (listed in the text). **B**, CPE-mediated increase in [^3^H] DA uptake by DAT was blocked with the coexpression of a minigene encoding the DAT-CT583–620 peptide but did not alter the uptake of cells expressing DAT alone (n = 4, P < 0.01). **C**, Representative curves of the dose-dependent inhibition of [^3^H] WIN (4 nM) binding by dopamine to DAT. Coexpression of CPE and DAT did not alter the estimated affinity value of dopamine for DAT (IC50 values listed in the text). **D**, Estimated IC50 values in control or CPE coexpressing cells. Values represent the means ± SEM of 3–9 independent experiments, each conducted in duplicate. Data were analyzed by ANOVA, followed by *post hoc *Student-Newman-Keuls test. **, P < 0.01 compared with the group cotransfected with DAT and pcDNA3; ##, P < 0.01 compared with the group cotransfected with DAT and CPE.

DAT plays a determinant role in dopaminergic neurotransmission by rapidly removing released dopamine from the synaptic cleft. To explore the functional importance of the DAT-CPE interaction, we examined the DAT-mediated DA uptake activity in HEK-293 cells coexpressing CPE. As illustrated in Fig 2A, CPE coexpression significantly increased the maximal uptake velocity (Vmax) of [^3^H] DA by ~50% (DAT: Vmax = 10.8 ± 1 pmol/min/well; DAT+CPE: 16 ± 1.9 pmol/min/well; *P *< 0.01), whereas no significant change in the estimated *Km *of the DAT could be observed (DAT: *Km *= 6.6 ± 0.7 μM in cells expressing DAT vs 6.7 ± 1.6 μM in cells expressing DAT and CPE). To further exclude the possibility that Vmax differences might result from the affinity changes, we performed [^3^H] WIN 35,428 competitive binding assay. As depicted in Fig. 2C and 2D, the estimated affinity of DA at the ligand binding site of DAT in CPE coexpressing cells was not significantly different from that observed for cells expressing DAT alone (*IC*_50 _= 986 ± 24 nM in cells expressing DAT vs 1001 ± 87 nM in cells expressing DAT and CPE). The effect of CPE on DAT was not restricted to HEK-293 cells but was also seen in COS-7 and Ltk- cells (data not show).

Next, to confirm that the selective increase in maximal DAT-mediated DA uptake resulted from DAT-CPE interaction, we tested whether a minigene (MG) encoding sequences of the DAT-CT could block these effects, since coexpressioin of CT tail peptide would act as a competitive inhibitor to the CPE-DAT interaction. As described in Fig. 2B, DAT-CT tail583–620 MG cotransfected with DAT alone did not change either the Km or Vmax of cellular [^3^H] DA uptake. However, coexpression of the MG encoding the peptide DAT-CT583–620 significantly inhibited the increasing effects of DAT-mediated DA translocation by CPE. Taken together, these data indicated that the binding of DAT-CT tail583–620 sequences to CPE was both sufficient and necessary for the expression of CPE- mediated functional regulation of the DA translocation process.

### Regulation of DAT expression and trafficking by CPE

**Figure 3:**
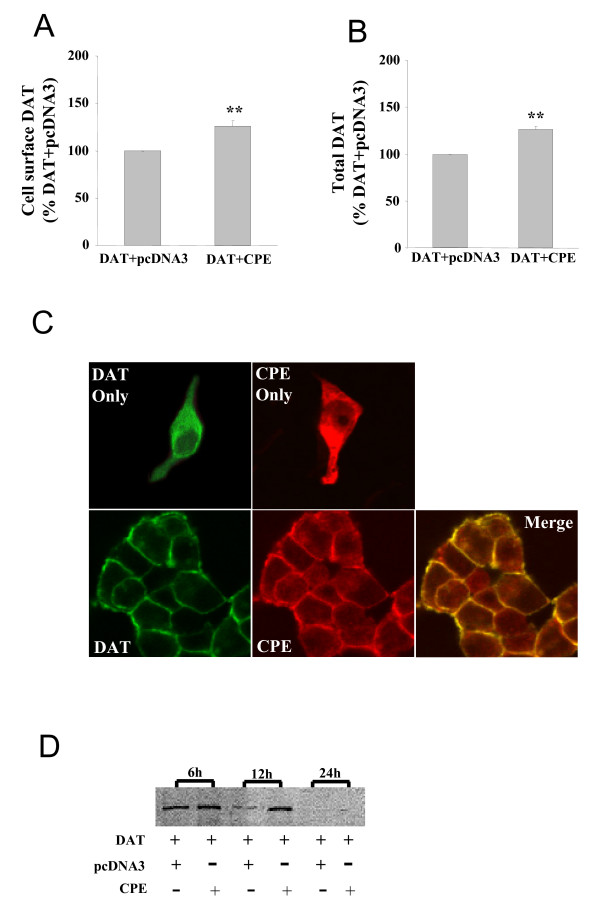
**A, Summarized data indicating effects of CPE on DAT membrane expression in cotransfected HEK-293 cells**. Columns show the means ± SEM of the ratios of colorimetric readings under coexpression of DAT and pcDNA3 conditions versus coexpression of DAT and CPE. **P < 0.01, n = 3 (Student's *t *test). **B**, Summarized data indicating effects of CPE on DAT total expression including the extracellular and intracellular ones in cotransfected HEK-293 cells. Columns show the means ± SEM of the ratios of colorimetric readings under coexpression of DAT and pcDNA3 conditions versus coexpression of DAT and CPE. **P < 0.01, n = 3 (Student's *t *test). **C**, CPE colocalizes with DAT in HEK-293 cells. The distribution pattern of DAT and CPE when expressed individually or coexpressed in HEK-293 cells are shown. Immunostaining was performed using the rabbit anti-DAT or the mouse anti-CPE antibodies and secondary antibodies:CY3 anti-mouse (red) and FITC-conjugated anti-rabbit antibodies (green). Yellow staining on the merged panels depicted colocalization. No staining was observed when primary antibodies were eliminated. Data are representative of 4–6 independent experiments, with a total cell sampling of more than 1000. **D**, Measurement of DAT protein metabolism by labeling with radioactive amino acids. HEK-293 cells, into which the indicated genes had been transfected, acutely incorporated 0.2 mCi/ml promix [^35^S]-methionine/cysteine for 4 h and were grown in the complete medium for further 6, 12 and 24 h. DAT protein was immunoprecipitated from the total protein by an anti-DAT polyclonal antibody. The antigen-antibody complex was further analyzed on polyacrylamide gel and autoradiography was performed. The results were repeated 3 times.

Since no change in DA uptake affinity could be observed upon coexpression of CPE with DAT, the increase in Vmax may reflect a change in the population of cell surface DAT. Therefore, we next examined if DAT cell surface expression levels change upon coexpression of CPE by using a cell-based ELISA assay to quantify changes at the cell surface and total DAT levels [27-29]. As shown in Fig 3, coexpression of CPE increased the levels of both total DAT and cell surface DAT to about ~20% (Fig. 3A, B).

To directly examine the cellular effects of CPE on DAT, we analyzed subcellular localization of DAT and CPE in transfected HEK-293 cells using confocal immunofluorescence microscopy. The results showed that DAT and CPE displayed disperse distribution patterns when expressed separately in HEK-293 cells. DAT could be found both at the cell surface and within cell, whereas CPE located throughout the cytoplasm (Fig. 3A, B). However, when CPE and DAT were cotransfected in HEK-293 cells, the distribution pattern of each protein changed dramatically, with almost completely overlapping distribution at cell surface, as revealed by the merged confocal immunofluorescence images (Fig. 3C). In virtually all cells coexpressing DAT and CPE, DAT was largely found at the plasma membrane and colocalized with CPE (Fig. 3C). These data suggested that CPE modulated DAT via a posttranslational mechanism, which possibly included reduced degradation of surface DAT.

Cell surface DAT may derive from newly synthesized DAT or from recycled DAT that have been re-inserted into the membrane after being internalized. Thus, changes in both cell surface and total DAT levels may reflect changes in DAT protein turnover rates in the presence of CPE. To test this possibility, we carried out pulse-chase studies to examine DAT protein half-life in the presence of CPE. As showed in Fig. 3D, in the absence of CPE, the DAT-positive band was prominent at 6 hours but began to disappear at 12 hours and was completely absent at 24 hours. However, coexpression of CPE inhibited the disappearance of the DAT-positive band at 12 hours but was still completely degraded at 24 hours.

### CPE stablized DAT via reducing the phosphorylation state of DAT

**Figure 4:**
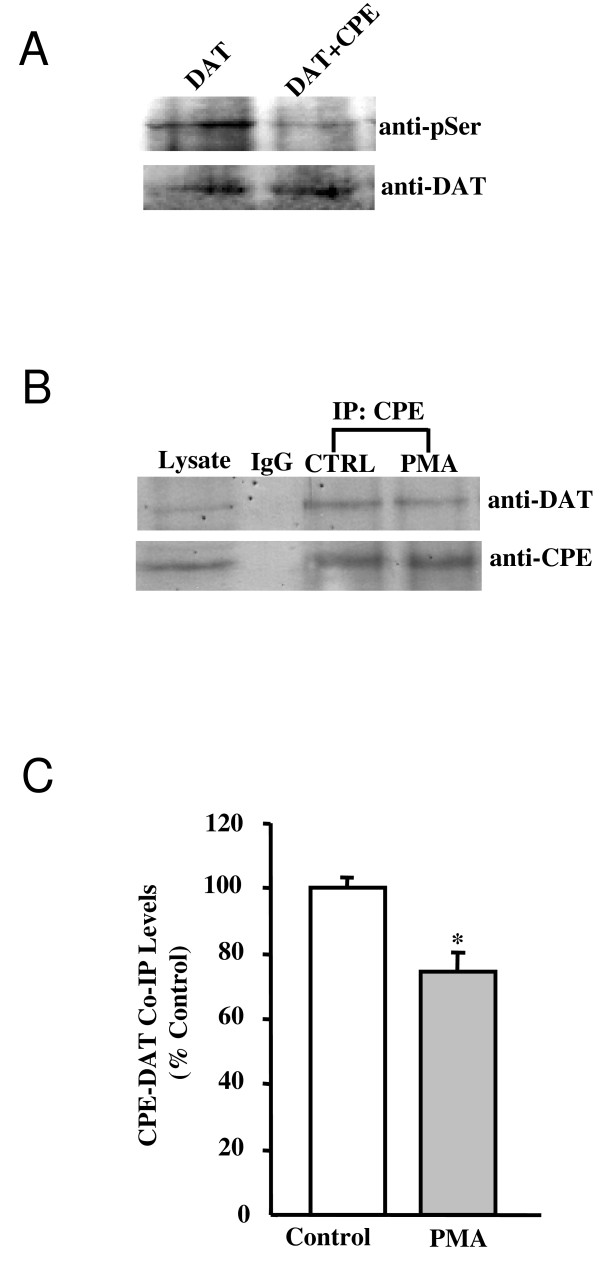
**CPE affects the phosphorylation state of DAT**. **A**, COS-7 cells were transiently transfected with DAT or together with CPE by using electroporation, grown for 3–4 days, and was collected to be homogenized. The cells lysates was immunoprecipitated with anti-DAT antibody overnight. Subsequent western blotting of SDS-PAGE immunoprecipitaed samples with polyclonal anti-phosphoserine antibody and reimmunoblotting the membrane with anti-DAT antibody. **B**, Phosphorylation state of DAT affected the interaction between DAT and CPE. Striatal slices were pretreated for 30 min with 2 μM PMA before immunoprecipitation with the DAT antibody. Subsequent western blotting of SDS-PAGE immunoprecipitaed samples were incubated with anti DAT or anti CPE antibody.

Cell surface expression and degradation of DAT [31] is modulated by various cellular signals, substrates and neuronal activity. Among them, the best characterized form of DAT regulation is phosphorylation [32]. Previous reports show that protein kinase C (PKC) down-regulates transporter activity via internalization of DAT in several systems including striatal synaptosomes [33,34], slices [35] and heterologous cells [31,36,37]. Thus, the increased surface expression of DAT by CPE may involve phosphorylation events. To explore this possibility, we first tested whether CPE had any effect on the phosphrylation levels of DAT. As illustrated in Fig. 4A, using an antibody that detected phosphorylated serines, immunoprecipitation of DAT from COS-7 cells revealed a decrease in serine phosphorylation in cells coexpressing CPE. Reduced phosphorylation state of DAT might reflect that association of CPE with DAT leaded to the occlusion of PKC phosphorylation sites. We then examined the association between CPE and DAT under various phosphorylation conditions. Rat striatum slices were treated with a PKC activator, phorbol 12-myristate 13-acetate (PMA) for 30 min before immunoprecipitation with the CPE antibody. As shown in Fig 4B, PMA treatment reduced the association between CPE and DAT as compared with non-treated COS-7 cells, implying that CPE preferentially interacted with non-PKC phosphorylated forms of DAT.

### CPE modulated the function of DAT through surface phosphorylation and expression

**Figure 5:**
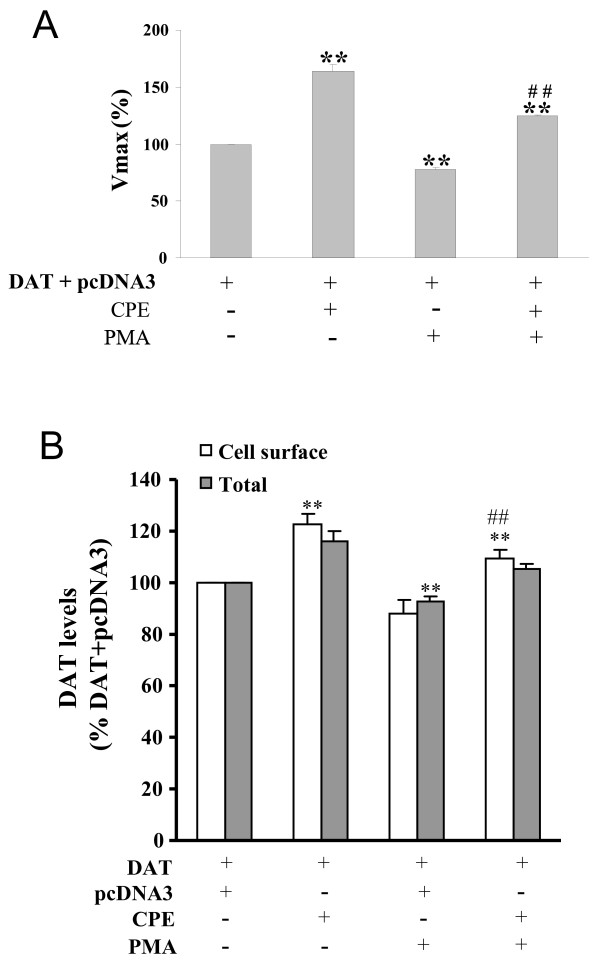
**CPE reserves PKC-mediated inhibition of DAT uptake and cell surface expression**. **A**, Uptake of [^3^H] DA in HEK-293 cells after a 30-min incubation at 37°C with vehicle alone (uptake buffer) or 2 μM PMA. Values are the percentage of uptake activity compared with untreated cells ± SEM. Data were analyzed by ANOVA, followed by *post hoc *Student-Newman-Keuls test. **, P < 0.01 compared with the group cotransfected with DAT and pcDNA3; ##, P < 0.01 compared with the group cotransfected with DAT and CPE untreated with PMA, n = 3. **B**, Summarized data indicating effects of CPE on DAT membrane expression in cotransfected HEK-293 cells after 30-min incubation at 37°C with vehicle alone (uptake buffer) or 2 μM PMA. Columns show the means ± SEM of the ratios of colorimetric readings. Data were analyzed by ANOVA, followed by *post hoc *Student-Newman-Keuls test. **, P < 0.01 compared with the group cotransfected with DAT and pcDNA3; ##, P < 0.01 compared with the group cotransfected with DAT and CPE untreated with PMA, n = 3.

To provide further evidence that the expression and functional affects on DAT were mediated by phosphorylation changes induced by the CPE-DAT interaction, we explored the relationship of DAT phosphorylation with DA translocation velocity and surface expression under phosphorylating conditions mentioned above. As depicted in Fig 5A, PMA treatment significantly reduced the increasing effects of CPE on DAT-induced DA translocation velocity. Moreover, the changes of Vmax levels coincided with both the total and surface expression levels of DAT (Fig 5B); strongly suggesting that PMA reduced the effects of CPE probably through increased internalization via phosphorylation.

## Discussion

Recent studies have identified new properties of the DAT, including the regulation of cell excitability through leak current conductance and channel mechanism [38,39], as well as serving as a pathway for substrate-induced dopamine release at both axonal and dendritic sites [40]. However, each cellular role currently associated with the DAT depends on plasma membrane expression. In the present study, we provided biochemical evidence for a physical interaction between CPE and DAT via the DAT-CT. We have shown that this interaction leads to cell surface stabilization of DAT via reduced degradation, possibly through a reduction in DAT phosphorylation. In addition, immunofluorescence analysis demonstrated that co-expression of CPE and DAT facilitated surface translocation of both proteins. Functional assays showed that the increased surface DAT led to a corresponding increase in DA uptake but had no effect on DAT affinity for DA. Finally, mini-gene encoding the DAT CT blocked CPE's effects. We concluded that CPE-mediated modulation of DAT trafficking, membrane expression and phosphorylation was facilitated by the CPE-DAT physical coupling.

The present finding highlighted the importance of CPE in trafficking and metabolic processing of DAT. DAT undergoes cyclical traffic to and from the plasma membrane and changes in transporter surface presentation can result from modulating endocytosis, recycling or de novo biosynthesis. Coexpression of CPE with DAT changed the diffuse DAT expression throughout the cell to stronger plasma membrane localization and suggests the interaction occur intracellularlly. CPE exists in both a soluble form and a membrane form [41,42]. Soluble forms of CPE function as an exopeptidase to cleave proneuropeptides within the lumen of the vesicle and yield biologically active peptides [43,44], whereas membrane from of CPE is associated with lipid rafts and exist in a transmembrane orientation in secretory granules as a sorting receptor [45]. During proteolytic processing of peptide precursor, CPE normally removes the C-terminal basic residues on the processing intermediates after the action of prohormone convertases on the peptide precursor [46]. In many cases, as a final step in the biosynthesis of neuropeptides, CPE recognizes and cleaves the Arg/Lys paired basic residues at the COOH-terminal side to generate the final neuropeptide. Thus, the exact nature of CPE-DAT association, how and where this interaction takes place intracellularly remains to be determined.

Although not in paired form, Arg/Lys residues are also present in the C-terminus of hDAT, rendering the possibility that CPE would be involved in the processing of DAT. Interestingly, CPE band did show a slightly lower on pulse-chase assay coexpressed with DAT, which deserved further investigation. Immunogold labeling detects DAT in tubulo-vesicular structures and small electron-lucent vesicles in dopaminergic neurons from the dorsal striatum and nucleus accumbens [8-10]. Also, both ultrastructural and biochemical studies revealed the localization of DAT in endosomes and tubulo-vesicular membranes but not biosynthetic machinery, suggesting that most of intracellular DAT- positive vesicles are endocytic but not secretory [14,37]. Interestingly, the membrane form of CPE has been demonstrated to bind to POMC [30] and BDNF [41], sorting these precursors at the trans-Golgi network (TGN) into vesicles of the regulated secretory pathway in pituitary cells and hippocampal neurons, respectively. Previous results show that secretory and endocytic pathways converge forming the TGN, an intricate system of tubulovesicular membranes, the main site for sorting secretory vesicles and transports proteins including DAT [9,11,47,48] and for specifically targeting synaptic membrane receptors into dendrites [49]. Moreover, the TGN is known to interact with the recycling endosomal system in hippocampal neurons [50]. Thus, the co-localization of these two proteins in TGN may be the potential site where the CPE-DAT interaction occurs.

DAT possesses consensus sequences for phosphorylation by protein kinases [6,13,51,52] including cAMP-dependent protein kinsase, protein kinase C (PKC), Ca2+/calmodulin-dependent protein kinase, MAP kinase and tyrosine kinase. Several studies have shown that regulation of kinase activity in striatal synaptosomal preparations and heterologous cell expression systems affects DAT uptake activity [12], which is believed to result from a rapid redistribution of transporter from the cell membrane to intracellular compartments, rather than changes in the intrinsic transport activity [31,37,53,54]. This phosphorylation/dephosphorylation status is closely coupled to DAT endocytic trafficking and interestingly PKC activation has been shown to decrease DAT activity through clathrin-mediated endocytosis [31,55], an event associated with an increase in DAT phosphorylation [52]. This is in agreement with our studies in which CPE decreases DAT phosphorylation leading to slower degradation and increase cell surface localization. However, there have been reports that PKC-induced phosphorylation of the DAT itself is not required for PKC-induced DAT sequestration [56], leading to the possibility that DAT are trapped in its phosphorylated state in subcellular compartment, where CPE may speed up the assorting and recycling back to the membrane of the internalized DAT.

The exact mechanism for CPE's effects on DAT phosphorylation and its relationship with cell surface DAT expression require further investigation. Foster and colleagues recently report that the amino terminus of DAT, which contains a cluster of serine residues, is the primary site of phosphorylation by PKC [35]. It is possible that the interaction with CPE may mask the PKC phosphorylation site on DAT. Indeed, it has been proposed that transporter-interacting proteins might directly or indirectly regulate kinase-dependent effects on DAT activity [57]. Alternatively, there is evidence that PKC- mediated functional regulation of DAT is not achieved by direct phosphorylation of the DAT protein [58], as has been demonstrated for the GABA transporter, where PKC down regulates transporter function by modulating the association of syntaxin with the transporter [59]. Moreover, other yet-to-be-identified proteins may also play a role in regulating DAT function. Blakely and colleagues found that the protein phosphatase 2A (PP2A) could be co-immunoprecipitated from brain tissue with DAT, NET, or SERT proteins [60]. In the case of SERT, the interaction with PP2A was dynamically regulated by phosphatase inhibition, PKC activation and transporter substrates. Although it is not known whether PP2A binds directly to monoamine transporters, this association might help to explain the molecular events that are responsible for transporter phosphorylation and internalization

## Conclusion

Taken together, data from our present study and previous reports indicate that protein-protein interactions appear to be important for DAT function. The association of DAT with CPE may have critical impact on DAT phosphorylation state and be involved in cyclic trafficking. While our findings shed new light on the trafficking mechanisms of DAT, several questions still remain to be addressed. It remains to be determined in which cellular compartment this interaction occurs and the details of mechanisms involved that may regulate the CPE-DAT interaction. Furthermore, both CPE and the DAT may have roles in obesity and it will be interesting to investigate if this interaction contributes to the pathophysiology of this disease. Further investigation into characterizing this interaction and understanding the regulatory factors involved, will lead to a better understanding of DAT trafficking and may have important physiological and pathophysiological implications.

## Competing interests

The authors declare that they have no competing interests.

## Authors' contributions

HZ carried out all experiments, with the exception of the in vitro binding, the uptake assay, WIN binding and the assays of the phosphorylation level of DAT. SL conducted the immunoassays of the phosphorylation level of DAT and contribute to the writing of the manuscript. MW participated in the in vitro binding and helped to draft the manuscript. BV participated in the cell culture and performed the statistical analysis. ZBP provided the cDNA of the dopamine transporter and conducted the dopamine uptake and WIN binding assays. FL conceived of the study, and participated in its design and wrote the manuscript. All authors read and approved the final manuscript.

## References

[B1] Amara SG, Kuhar MJ (1993). Neurotransmitter transporters: recent progress. Annu Rev Neurosci.

[B2] Norregaard L, Gether U (2001). The monoamine neurotransmitter transporters: structure, conformational changes and molecular gating. Curr Opin Drug Discov Devel.

[B3] Gether U, Andersen PH, Larsson OM, Schousboe A (2006). Neurotransmitter transporters: molecular function of important drug targets. Trends Pharmacol Sci.

[B4] Chen N, Reith ME (2000). Structure and function of the dopamine transporter. Eur J Pharmacol.

[B5] Bannon MJ (2005). The dopamine transporter: role in neurotoxicity and human disease. Toxicol Appl Pharmacol.

[B6] Zahniser NR, Sorkin A (2004). Rapid regulation of the dopamine transporter: role in stimulant addiction?. Neuropharmacology.

[B7] Torres GE, Carneiro A, Seamans K, Fiorentini C, Sweeney A, Yao WD, Caron MG (2003). Oligomerization and trafficking of the human dopamine transporter. Mutational analysis identifies critical domains important for the functional expression of the transporter. J Biol Chem.

[B8] Nirenberg MJ, Vaughan RA, Uhl GR, Kuhar MJ, Pickel VM (1996). The dopamine transporter is localized to dendritic and axonal plasma membranes of nigrostriatal dopaminergic neurons. J Neurosci.

[B9] Nirenberg MJ, Chan J, Vaughan RA, Uhl GR, Kuhar MJ, Pickel VM (1997). Immunogold localization of the dopamine transporter: an ultrastructural study of the rat ventral tegmental area. J Neurosci.

[B10] Nirenberg MJ, Chan J, Pohorille A, Vaughan RA, Uhl GR, Kuhar MJ, Pickel VM (1997). The dopamine transporter: comparative ultrastructure of dopaminergic axons in limbic and motor compartments of the nucleus accumbens. J Neurosci.

[B11] Schroeter S, Apparsundaram S, Wiley RG, Miner LH, Sesack SR, Blakely RD (2000). Immunolocalization of the cocaine- and antidepressant-sensitive l-norepinephrine transporter. J Comp Neurol.

[B12] Zahniser NR, Doolen S (2001). Chronic and acute regulation of Na+/Cl- -dependent neurotransmitter transporters: drugs, substrates, presynaptic receptors, and signaling systems. Pharmacol Ther.

[B13] Gulley JM, Zahniser NR (2003). Rapid regulation of dopamine transporter function by substrates, blockers and presynaptic receptor ligands. Eur J Pharmacol.

[B14] Melikian HE (2004). Neurotransmitter transporter trafficking: endocytosis, recycling, and regulation. Pharmacol Ther.

[B15] Lee FJ, Pristupa ZB, Ciliax BJ, Levey AI, Niznik HB (1996). The dopamine transporter carboxyl-terminal tail. Truncation/substitution mutants selectively confer high affinity dopamine uptake while attenuating recognition of the ligand binding domain. J Biol Chem.

[B16] Torres GE, Yao WD, Mohn AR, Quan H, Kim KM, Levey AI, Staudinger J, Caron MG (2001). Functional interaction between monoamine plasma membrane transporters and the synaptic PDZ domain-containing protein PICK1. Neuron.

[B17] Carneiro AM, Ingram SL, Beaulieu JM, Sweeney A, Amara SG, Thomas SM, Caron MG, Torres GE (2002). The multiple LIM domain-containing adaptor protein Hic-5 synaptically colocalizes and interacts with the dopamine transporter. J Neurosci.

[B18] Lee FJ, Liu F, Pristupa ZB, Niznik HB (2001). Direct binding and functional coupling of alpha-synuclein to the dopamine transporters accelerate dopamine-induced apoptosis. FASEB J.

[B19] Sorkina T, Doolen S, Galperin E, Zahniser NR, Sorkin A (2003). Oligomerization of dopamine transporters visualized in living cells by fluorescence resonance energy transfer microscopy. J Biol Chem.

[B20] Lee KH, Kim MY, Kim DH, Lee YS (2004). Syntaxin 1A and receptor for activated C kinase interact with the N-terminal region of human dopamine transporter. Neurochem Res.

[B21] Bolan EA, Kivell B, Jaligam V, Oz M, Jayanthi LD, Han Y, Sen N, Urizar E, Gomes I, Devi LA (2007). D2 receptors regulate dopamine transporter function via an extracellular signal-regulated kinases 1 and 2-dependent and phosphoinositide 3 kinase-independent mechanism. Mol Pharmacol.

[B22] Lee FJ, Pei L, Moszczynska A, Vukusic B, Fletcher PJ, Liu F (2007). Dopamine transporter cell surface localization facilitated by a direct interaction with the dopamine D2 receptor. EMBO J.

[B23] Marazziti D, Mandillo S, Di Pietro C, Golini E, Matteoni R, Tocchini-Valentini GP (2007). GPR37 associates with the dopamine transporter to modulate dopamine uptake and behavioral responses to dopaminergic drugs. Proc Natl Acad Sci USA.

[B24] Zapata A, Kivell B, Han Y, Javitch JA, Bolan EA, Kuraguntla D, Jaligam V, Oz M, Jayanthi LD, Samuvel DJ (2007). Regulation of dopamine transporter function and cell surface expression by D3 dopamine receptors. J Biol Chem.

[B25] Cen X, Nitta A, Ibi D, Zhao Y, Niwa M, Taguchi K, Hamada M, Ito Y, Wang L, Nabeshima T (2008). Identification of Piccolo as a regulator of behavioral plasticity and dopamine transporter internalization. Mol Psychiatry.

[B26] Torres GE (2006). The dopamine transporter proteome. J Neurochem.

[B27] Lee FJ, Xue S, Pei L, Vukusic B, Chery N, Wang Y, Wang YT, Niznik HB, Yu XM, Liu F (2002). Dual regulation of NMDA receptor functions by direct protein-protein interactions with the dopamine D1 receptor. Cell.

[B28] Liu F, Wan Q, Pristupa ZB, Yu XM, Wang YT, Niznik HB (2000). Direct protein-protein coupling enables cross-talk between dopamine D5 and gamma-aminobutyric acid A receptors. Nature.

[B29] Pei L, Lee FJ, Moszczynska A, Vukusic B, Liu F (2004). Regulation of dopamine D1 receptor function by physical interaction with the NMDA receptors. J Neurosci.

[B30] Cool DR, Normant E, Shen F, Chen HC, Pannell L, Zhang Y, Loh YP (1997). Carboxypeptidase E is a regulated secretory pathway sorting receptor: genetic obliteration leads to endocrine disorders in Cpe(fat) mice. Cell.

[B31] Daniels GM, Amara SG (1999). Regulated trafficking of the human dopamine transporter. Clathrin-mediated internalization and lysosomal degradation in response to phorbol esters. J Biol Chem.

[B32] Eisenhofer G (2001). The role of neuronal and extraneuronal plasma membrane transporters in the inactivation of peripheral catecholamines. Pharmacol Ther.

[B33] Copeland BJ, Vogelsberg V, Neff NH, Hadjiconstantinou M (1996). Protein kinase C activators decrease dopamine uptake into striatal synaptosomes. J Pharmacol Exp Ther.

[B34] Vaughan RA, Huff RA, Uhl GR, Kuhar MJ (1997). Protein kinase C-mediated phosphorylation and functional regulation of dopamine transporters in striatal synaptosomes. J Biol Chem.

[B35] Foster JD, Pananusorn B, Vaughan RA (2002). Dopamine transporters are phosphorylated on N-terminal serines in rat striatum. J Biol Chem.

[B36] Huff RA, Vaughan RA, Kuhar MJ, Uhl GR (1997). Phorbol esters increase dopamine transporter phosphorylation and decrease transport Vmax. J Neurochem.

[B37] Melikian HE, Buckley KM (1999). Membrane trafficking regulates the activity of the human dopamine transporter. J Neurosci.

[B38] Carvelli L, McDonald PW, Blakely RD, Defelice LJ (2004). Dopamine transporters depolarize neurons by a channel mechanism. Proc Natl Acad Sci USA.

[B39] Ingram SL, Prasad BM, Amara SG (2002). Dopamine transporter-mediated conductances increase excitability of midbrain dopamine neurons. Nat Neurosci.

[B40] Sulzer D, Maidment NT, Rayport S (1993). Amphetamine and other weak bases act to promote reverse transport of dopamine in ventral midbrain neurons. J Neurochem.

[B41] Lou H, Kim SK, Zaitsev E, Snell CR, Lu B, Loh YP (2005). Sorting and activity-dependent secretion of BDNF require interaction of a specific motif with the sorting receptor carboxypeptidase e. Neuron.

[B42] Fricker LD, Das B, Angeletti RH (1990). Identification of the pH-dependent membrane anchor of carboxypeptidase E (EC 3.4.17.10). J Biol Chem.

[B43] Fricker LD, Snyder SH (1982). Enkephalin convertase: purification and characterization of a specific enkephalin-synthesizing carboxypeptidase localized to adrenal chromaffin granules. Proc Natl Acad Sci USA.

[B44] Hook VY, Loh YP (1984). Carboxypeptidase B-like converting enzyme activity in secretory granules of rat pituitary. Proc Natl Acad Sci USA.

[B45] Dhanvantari S, Arnaoutova I, Snell CR, Steinbach PJ, Hammond K, Caputo GA, London E, Loh YP (2002). Carboxypeptidase E, a prohormone sorting receptor, is anchored to secretory granules via a C-terminal transmembrane insertion. Biochemistry.

[B46] Fricker LD (1988). Carboxypeptidase E. Annu Rev Physiol.

[B47] Mellman I, Simons K (1992). The Golgi complex: in vitro veritas?. Cell.

[B48] Lippincott-Schwartz J, Roberts TH, Hirschberg K (2000). Secretory protein trafficking and organelle dynamics in living cells. Annu Rev Cell Dev Biol.

[B49] Trimmer JS (1999). Sorting out receptor trafficking. Neuron.

[B50] Maletic-Savatic M, Malinow R (1998). Calcium-evoked dendritic exocytosis in cultured hippocampal neurons. Part I: trans-Golgi network-derived organelles undergo regulated exocytosis. J Neurosci.

[B51] Mortensen OV, Amara SG (2003). Dynamic regulation of the dopamine transporter. Eur J Pharmacol.

[B52] Foster JD, Cervinski MA, Gorentla BK, Vaughan RA (2006). Regulation of the dopamine transporter by phosphorylation. Handb Exp Pharmacol.

[B53] Doolen S, Zahniser NR (2001). Protein tyrosine kinase inhibitors alter human dopamine transporter activity in Xenopus oocytes. J Pharmacol Exp Ther.

[B54] Pristupa ZB, McConkey F, Liu F, Man HY, Lee FJ, Wang YT, Niznik HB (1998). Protein kinase-mediated bidirectional trafficking and functional regulation of the human dopamine transporter. Synapse.

[B55] Sorkina T, Hoover BR, Zahniser NR, Sorkin A (2005). Constitutive and protein kinase C-induced internalization of the dopamine transporter is mediated by a clathrin-dependent mechanism. Traffic.

[B56] Granas C, Ferrer J, Loland CJ, Javitch JA, Gether U (2003). N-terminal truncation of the dopamine transporter abolishes phorbol ester- and substance P receptor-stimulated phosphorylation without impairing transporter internalization. J Biol Chem.

[B57] Blakely RD, Ramamoorthy S, Schroeter S, Qian Y, Apparsundaram S, Galli A, DeFelice LJ (1998). Regulated phosphorylation and trafficking of antidepressant-sensitive serotonin transporter proteins. Biol Psychiatry.

[B58] Chang MY, Lee SH, Kim JH, Lee KH, Kim YS, Son H, Lee YS (2001). Protein kinase C-mediated functional regulation of dopamine transporter is not achieved by direct phosphorylation of the dopamine transporter protein. J Neurochem.

[B59] Beckman ML, Bernstein EM, Quick MW (1998). Protein kinase C regulates the interaction between a GABA transporter and syntaxin 1A. J Neurosci.

[B60] Bauman AL, Apparsundaram S, Ramamoorthy S, Wadzinski BE, Vaughan RA, Blakely RD (2000). Cocaine and antidepressant-sensitive biogenic amine transporters exist in regulated complexes with protein phosphatase 2A. J Neurosci.

